# Use of Amniotic Membrane and Its Derived Products for Bone Regeneration: A Systematic Review

**DOI:** 10.3389/fbioe.2021.661332

**Published:** 2021-05-11

**Authors:** Marion Etchebarne, Jean-Christophe Fricain, Halima Kerdjoudj, Roberta Di Pietro, Susanne Wolbank, Florelle Gindraux, Mathilde Fenelon

**Affiliations:** ^1^Univ. Bordeaux, INSERM, BIOTIS, U1026, Bordeaux, France; ^2^CHU Bordeaux, Department of Maxillofacial Surgery, Bordeaux, France; ^3^CHU Bordeaux, Service de Chirurgie Orale, Bordeaux, France; ^4^Université de Reims Champagne Ardenne, EA 4691, Biomatériaux et Inflammation en Site Osseux (BIOS), Reims, France; ^5^Université de Reims Champagne Ardenne, UFR d'Odontologie, Reims, France; ^6^Department of Medicine and Ageing Sciences, Gabriele D'Annunzio University of Chieti-Pescara, Chieti, Italy; ^7^StemTeCh Group, Gabriele D'Annunzio Foundation, Gabriele D'Annunzio University of Chieti-Pescara, Chieti, Italy; ^8^Ludwig Boltzmann Institute for Experimental and Clinical Traumatology, AUVA Research Center, Vienna, Austria; ^9^Austrian Cluster for Tissue Regeneration, Vienna, Austria; ^10^Service de Chirurgie Orthopédique, Traumatologique et Plastique, CHU Besançon, Besançon, France; ^11^Laboratoire de Nanomédecine, Imagerie, Thérapeutique EA 4662, Université Bourgogne Franche-Comté, Besançon, France

**Keywords:** amniotic membrane, amniotic epithelial cells, amniotic mesenchymal stromal cells, bone, bone tissue engineering, regenerative medicine, natural scaffold

## Abstract

Thanks to their biological properties, amniotic membrane (AM), and its derivatives are considered as an attractive reservoir of stem cells and biological scaffolds for bone regenerative medicine. The objective of this systematic review was to assess the benefit of using AM and amniotic membrane-derived products for bone regeneration. An electronic search of the MEDLINE—Pubmed database and the Scopus database was carried out and the selection of articles was performed following PRISMA guidelines. This systematic review included 42 articles taking into consideration the studies in which AM, amniotic-derived epithelial cells (AECs), and amniotic mesenchymal stromal cells (AMSCs) show promising results for bone regeneration in animal models. Moreover, this review also presents some commercialized products derived from AM and discusses their application modalities. Finally, AM therapeutic benefit is highlighted in the reported clinical studies. This study is the first one to systematically review the therapeutic benefits of AM and amniotic membrane-derived products for bone defect healing. The AM is a promising alternative to the commercially available membranes used for guided bone regeneration. Additionally, AECs and AMSCs associated with an appropriate scaffold may also be ideal candidates for tissue engineering strategies applied to bone healing. Here, we summarized these findings and highlighted the relevance of these different products for bone regeneration.

## Introduction

Reconstruction of large bone defects is a public health issue and a clinical challenge in orthopedic, plastic, oral, and maxillofacial surgery (Tseng et al., 2008; Nauth et al., 2011). Over the years, different strategies leading to the replacement of missing bone have been employed. Autologous bone grafts remain the most commonly used procedure for bone defect treatment. However, their limited availability and the additional donor site morbidity restrict their clinical applications (Delloye et al., 2007). Tissue engineering and regenerative medicine thus emerged as an option to overcome the limitation of conventional tissue grafting. In the field of bone regenerative therapies, various strategies using bioactive membranes, growth factors and/or stem cells have been intensively proposed (Stahl and Yang, 2020).

In this context, the amniotic membrane (AM) has become a highly attractive and easily accessible source of bioactive biological tissue containing growth factors and stem cells (Tamagawa et al., 2004; Ilancheran et al., 2009). AM is the innermost layer of the placenta and represents the border of the amniotic cavity, containing the amniotic fluid and the fetus. The AM contains three layers: an epithelial layer (mainly single) in touch with the amniotic fluid, a basement membrane and an avascular stroma layer. Two cell types compose the AM: amniotic-derived epithelial cells (AECs) and mesenchymal stromal cells (AMSCs). They produce extra-cellular matrix, different cytokines, and growth factors (Mamede et al., 2012). They are also known to display immunomodulatory properties and possess a pluripotent potential (Tamagawa et al., 2004; Ilancheran et al., 2007; Parolini et al., 2008, 2009; Centurione et al., 2018). No teratoma formation was reported in the literature following AM-derived stromal cells administration *in vitro* and *in vivo* (Parveen, 2018; Li et al., 2019; Liu et al., 2021). Due to its large availability, its anti-inflammatory (Hao et al., 2000) and anti-fibrotic (Ricci et al., 2013) properties, its low immunogenicity (Kang et al., 2012a) as well as the presence of mesenchymal stromal cells (Parolini et al., 2009) and growth factors (Koizumi et al., 2000; Grzywocz et al., 2014), AM has been used in therapy as a useful biological dressing in medicine, especially in ophthalmology or dermatology, since 1910. Since then, some studies have shown that AM and amniotic membrane-derived products are suitable for tissue engineering applications (Toda et al., 2007; Farhadihosseinabadi et al., 2018; Ramuta and Kreft, 2018) especially in the field of bone regeneration. Indeed, AM and its derivatives have been assessed in pre-clinical and clinical studies to this end.

First, it has been demonstrated that AM has the ability to be osteodifferentiated in toto, thereby suggesting promising results using this membrane in its entirety for bone regeneration (Lindenmair et al., 2010). Some studies thus suggested its potential as a biological alternative to membrane commonly used for guided bone regeneration (i.e., xenogeneic collagen membrane or synthetic membrane) (Gindraux et al., 2017; Aprile et al., 2020). To this end, AM has been assessed in therapy using different strategies: alone or associated with a bone substitute, as a covering or filling material of a bone defect, fresh or preserved (Fénelon et al., 2018a). To further improve its potential for bone regenerative medicine applications, some pre-clinical studies also reported the use of AM seeded with various types of stem cells (Tsugawa et al., 2011; Akazawa et al., 2016; Takizawa et al., 2019). Depending on these various usage strategies of AM, its ability to favor bone healing is still discussed, thus requiring an overview of its potential to favor bone healing within a given mode of application.

Another alternative to bone grafting is represented by tissue engineering cell-based strategies, which are aimed at achieving new bone formation via biomaterials used in combination with multipotent cells associated or not with bioactive molecules (Stahl and Yang, 2020). Stem cells are thus one of the most crucial components to fabricate these complex living constructs, mainly by seeding these cells on an appropriate scaffold matrix, on which they will grow and differentiate (Cancedda et al., 2003). AECs and AMSCs can be successfully isolated from AM and their osteogenic differentiation potential have been well-established *in vitro* (Ilancheran et al., 2007; Parolini et al., 2008, 2009; Díaz-Prado et al., 2010; Leyva-Leyva et al., 2013; Centurione et al., 2018). The ability of AECs or AMSCs associated to a scaffold, namely a bone substitute, to improve bone regeneration has thus been assessed *in vivo* (Tsuno et al., 2012; Barboni et al., 2013; Jiawen et al., 2014; Rameshbabu et al., 2016). However, there is no study summarizing techniques used to regenerate bone based on amniotic cells regenerative therapies.

Actually, there is no consensus regarding the optimal usage strategies of AM and its derivatives to promote bone healing. Thus, the objective of this review was to assess the potential of AM and its derivatives for bone regeneration and summarize how they should be used.

## Materials and Methods

This systematic review was performed according to the Preferred Reporting Items for Systematic Reviews and Meta-Analyses (PRISMA) (Swartz, 2011) and registered on PROSPERO database (N° CRD42019146785).

### Focused Question

The following focused question was defined: “What are the best strategies of using AM or AM-derived products in the field of bone regeneration?”

### Selection Criteria

All *in vivo* pre-clinical and clinical studies involving the AM or AM cells for bone regeneration were included. Only studies published in English with their abstracts available on database were considered. *In vitro* studies, case reports and systematic reviews were excluded. Moreover, studies in which the AM was not separated from the chorion were also excluded.

### Search Strategy

An electronic search was conducted on MEDLINE-PubMed and SCOPUS databases, for articles published in English, up to March 2021. The following search combination was used: (“amnion” OR “amniotic membrane” OR “amniotic cells”) AND (bone) AND (“*in vivo*” OR “pre-clinical” OR “clinical”). The reference lists of all publications selected were manually screened and additional articles complemented the electronic search.

### Screening Methods and Data Extraction

Two independent reviewers (ME and MF) performed the article selection and data extraction. Titles and abstracts were first screened according to the question: “What are the best strategies of using AM or AM-derived products in the field of bone regeneration?” If the titles and abstracts answered this screening question, full-text articles were then assessed. In case of disagreement between the reviewers, articles were discussed to decide the final outcome with the help of a third reviewer (FG). Relevant information of each article was collected in the data extraction tables, such as: general characteristics (authors and year of publication), the species, the model used, the type of amniotic products (membrane, stromal cells, and commercial products) and their characteristics. We also recorded treatment procedures, evaluation criteria, and the outcomes of included studies. For missing data, the authors were contacted by email to complete the information of the selected articles.

### Analysis of the Data

Data analysis was performed in a descriptive way, since the information obtained did not enable meta-analyses.

## Results

### Search Outcomes

**Figure 1:**
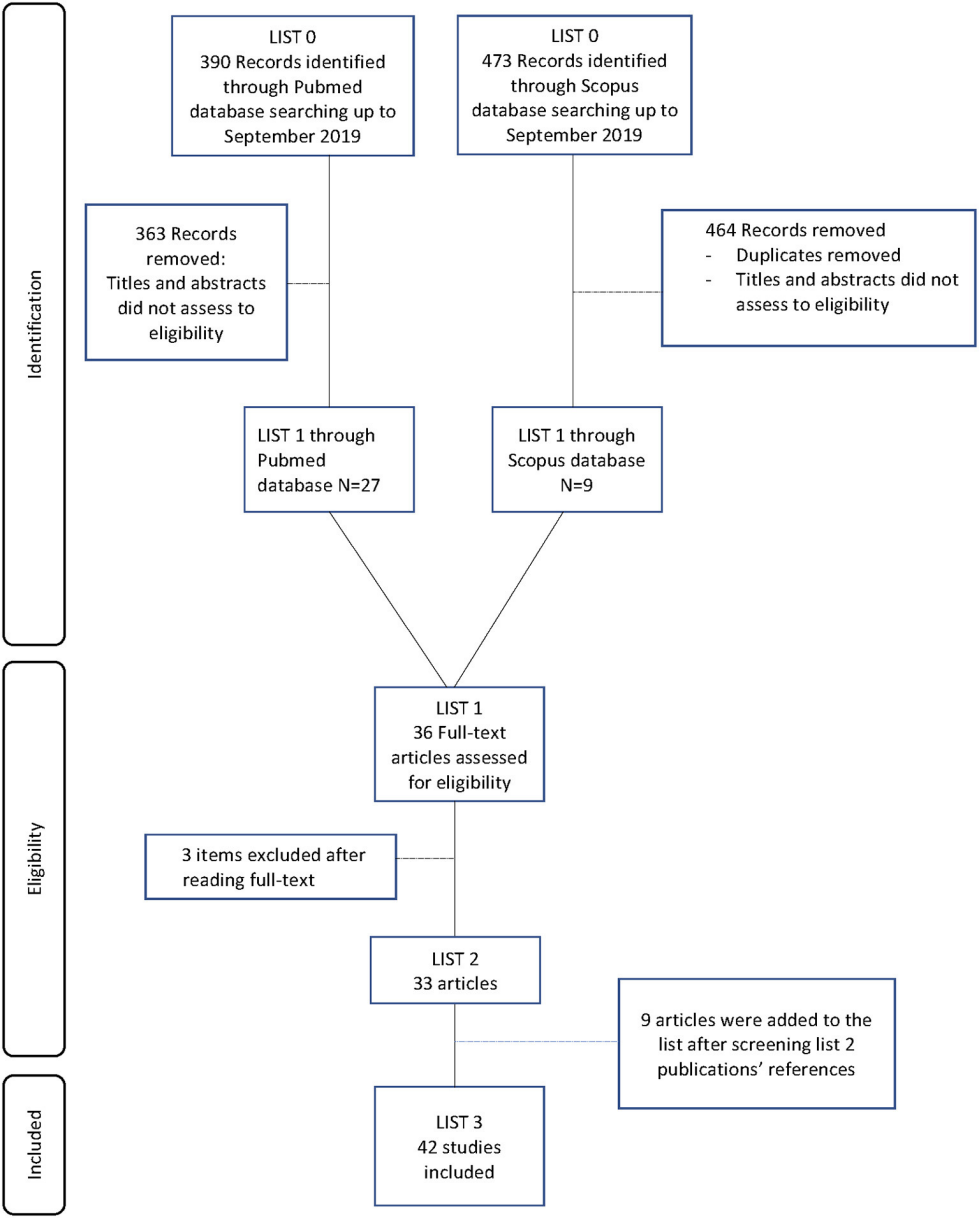
Flow diagram of the screened publications.

The electronic search generated 390 articles from MEDLINE-PubMed database and 473 articles from Scopus database. After reading the titles and abstracts, 36 articles were selected for further investigation. The entire publications were read and three articles were excluded. Nine articles were then added after manual literature searches (Figure 1). Finally, 42 studies met the eligibility criteria and were included to be analyzed in this systematic review. Twenty-one pre-clinical studies reported the use of AM for bone regeneration, while 10 other studies were focused on AM-derived stromal cells. We also identified seven clinical studies which investigated human AM therapeutic benefits for bone regeneration. Finally, four studies investigated the potential of commercialized products derived from AM for bone regeneration.

### Use of AM to Promote Bone Regeneration in Animal Models

Among the 21 pre-clinical studies investigating the effectiveness of AM for bone regeneration, two studies assessed its osteoinductive potential using an ectopic bone formation model (i.e., subcutaneous implantation), while all the remaining studies were conducted on orthotopic models. We also identified several AM usage strategies (Table 1). Most of the included studies used human AM, whereas two animal studies were performed with AM derived from rabbit or dog placenta. AM was either applied over the bone defect or implanted as a filling material inside the bone defect. Besides, AM was used either fresh (*n* = 5) or preserved with different methods such as cryopreservation (*n* = 8), lyophilization (*n* = 3), de-epithelialization (*n* = 2), and decellularization (*n* = 7). Finally, AM was mainly used alone or in association with a bone substitute (*n* = 13) (Table 2). Otherwise, AM was seeded with stromal cells before being implanted in animal models (*n* = 8) (Table 3).

**Table 1:** AM usage strategies to treat bone defects in preclinical and clinical studies.

**References**	**AM origin**	**AM preservation methods**	**Number of layer**	**AM disposition**	**AM uses**
**Pre-clinical studies**					
Gomes et al. (2001)	Human	Lyophilized	2	On the base and over the defect	Alone or combined with a bone substitute
Samandari et al. (2011)	Human	Cryopreserved	1	Over the defect	Alone
Li et al. (2015)	Human	Decellularized + Lyophilized	8	Over the defect	Covering a bone substitute
Fénelon et al. (2018b)	Human	Fresh or Cryopreserved	1	Over the defect	Alone
		Cryopreserved	1	Over the defect	Covering a bone substitute
Tang et al. (2018)	Human	De-epithelialized + Lyophilized	1	Over the defect	Alone
Ghanmi et al. (2018)	Human	Fresh	1	Over the defect	Alone
			3	Into the defect	
Khalil and Melek (2018)	Human	Lyophilized	1	Into the defect	Alone
Koushaei et al. (2018)	Human	Cryopreserved	1	Over the defect	Alone
Fenelon et al. (2020)	Human	Fresh/or Cryopreserved/or Lyophilized/or Decellularized + Lyophilized	1	Over the defect	Alone
Moosavi et al. (2018)	Human	Fresh	1	Over the defect *or* Into the defect	Alone or covering a bone substitute
Li W. et al. (2019)	Human	Decellularized	2	Over the defect	Alone or combined with a polymer
Sar et al. (2019)	Human	Cryopreserved	1	Over the defect	Alone
Tsugawa et al. (2011)	Human	De-epithelialized	1	Over the defect	Alone or seeded with cells
Semyari et al. (2016)	Rabbit	Decellularized	1	Over the defect	Alone or seeded with cells
Amer et al. (2015)	Dog	Cryopreserved	1	Into the defect	Alone or seeded with cells
Wu et al. (2016)	Human	De-epithelialized	1	Into the defect	Alone or seeded with cells
Akazawa et al. (2016)	Human	Decellularized	1	Over the defect	Seeded with cells
Takizawa et al. (2019)	Human	De-epithelialized	1	Into the defect	Seeded with cells
Sabouri et al. (2020)	Human	Decellularized	1	Over the defect	Alone or seeded with cells
Dziedzic et al. (2021)	Human	Decellularized	4	Into the defect	Alone or seeded with cells
**Clinical studies**					
Kothiwale et al. (2009)	Human	Lyophilized	1	Over the defect	Covering a bone substitute
Kiany and Moloudi (2015)	Human	Lyophilized	2	Over the defect	Covering a bone substitute
Kumar et al. (2015)	Human	Lyophilized	1	Over the defect	Covering a bone substitute
Sali and Pauline George (2016)	Human	Lyophilized	1	Over the defect	Covering a bone substitute
Pajnigara et al. (2017)	Human	Lyophilized	1	Over the defect	Covering a bone substitute
Kaur and Bathla (2018)	Human	Lyophilized	1	Over the defect	Covering a PRF membrane
Akhlaghi et al. (2019)	Human	Decellularized + Lyophilized	1	Over the defect	Covering a bone substitute

*PRF, platelet-rich fibrin*.

**Table 2:** Pre-clinical studies using AM alone and/or combined with a bone substitute for bone regeneration.

**References**	**Animal (No. per condition and per time)**	**Model: Defect localization and size**	**Treatments**	**Evaluation methodology**	**Results**
Laurent et al. (2017)	Mice (No. = 3)	Subcutaneous implantation (ectopic model)	**1st experimentation:** 1) AM 2) AM cultured in basal medium 3) Osteodifferentiated AM 4) Human skin **2nd experimentation:** 1) MBCP 2) MBCP + AM 3) MBCP + Osteodifferentiated AM	Histology	No ectopic bone formation was observed in any of the tested conditions for both experimentations after 1, 2, 4, and 8 weeks.
Samandari et al. (2011)	Dog (No. = 20)	Mucoperiosteal defect in jaw7 × 5 cm^2^	1) Orabase® dressing 2) AM covered by Orabase® dressing	Histology	AM significantly increased bone remodeling after 2, 8, and 12 weeks.
Tang et al. (2018)	Rat (No. = 15)	Femoral defect2.2 × 2.5 mm	1) Empty defect 2) AM	HistologyQuantitative real-time PCR	AM significantly increased bone formation after 15 and 30 days. AM induced a significant higher expression of gene connected with cell recruitment and bone remodeling.
Koushaei et al. (2018)	Dog (No. = 6, No. = 5, and No. = 8)	Tibial defectDiameter: 16 mm	1) Empty defect 2) Collagen membrane 3) AM	Histology	AM significantly increased bone formation compared to the defect left empty after 6 and 12 weeks. No significant differences between collagen membrane and the defect left empty.
Ghanmi et al. (2018)	Rabbit (No. = 5)	Tibial segmental defect20 mm	1) Empty defect (periosteum +) 2) Empty defect (periosteum –) 3) AM over the defect (periosteum –) 4)AM into the defect (periosteum –)	2D X-rays3D X-raysHistology	AM implanted over the defect group increased significantly bone regeneration compared to groups 1 and 4 after 4 and 8 weeks. Bone healing was even more promoted by the natural periosteum.
Khalil and Melek (2018)	Rabbit (No. = 6)	Femoral defect4 × 5 mm	1) Empty defect 2) AM into the defect	Histology	Woven bone was observed as early as the second week in the empty defect, whereas AM defect was filled by fibrous tissue and AM. Bone formation was significantly reduced in AM group after 2, 4, and 6 weeks compared to the empty group.
Sar et al. (2019)	Rat (No. = 7)	Tibial defectShaft fracture	1) Fracture fixation 2) Fracture fixation and AM wrapping	2D X-raysHistology	AM increased significantly bone formation and callus diameters after 3rd and 6th weeks.
Fénelon et al. (2018b)	Mice (No. = 8)	Calvarial defectDiameter: 3.3 mm	**1st experimentation:** 1) Empty defect 2) Fresh AM MES 3) Cryo AM MES 4) Fresh AM EPI 5) Cryo AM EPI		
			**2nd experimentation:** 1) HA 2) HA + Collagen membrane 3) HA + Cryo AM MES 4) HA + BMP2 membrane 5) HA + BMP2 + Collagen 6) HA + BMP2 + Cryo AM MES	2D X-raysHistology	Cryopreserved AM with mesenchymal side in contact with the defect increased significantly bone formation after 8 weeks. No significant differences between HA group, HA + AM group and HA + collagen membrane group after 6 weeks.
Gomes et al. (2001)	Rabbit (No. = 3)	Calvarial defect10 × 5 mm	1) AM 2) ADDM + AM	Histology	Newly formed bone was observed in both groups after 30 days. Mature bone tissue was observed in both groups after 120 days. Bone healing was faster in ADDM + AM group.
Li et al. (2015)	Rat (No. = 6)	Tibial defect2 × 2 × 2.5 mm	1) No defect 2) Empty defect 3) Bio-oss® 4) Bio-oss®+ collagen membrane 5) Bio-oss®+ AM	2D X-rays3D X-raysHistology	The gray level of the collagen and the AM groups were significantly higher than Bio-oss® group after 6 weeks. No significant differences were found between the AM group and the no defect group. A better bone–implant connection was also evidenced in the AM group.
Moosavi et al. (2018)	Rabbit (No. = 10)	Radius segmental defect15 mm	1) Empty defect 2) AM into the defect 3) AM over the defect 4) DBM + AM over defect	2D X-raysHistology	Bone formation was observed when AM was implanted over the defect whereas group 1 and 2 showed no bone compared to the three other groups after 8 weeks. A small amount of bone was evidenced filling the defect with DBM + AM.
Li W. et al. (2019)	Rat (No. = 5)	Cleft palate defect1.3 × 7 mm	1) No surgery 2) Empty defect 3) AM 4) AM-POC	Maxillary second molar widths3D X-raysHistology	AM improved significantly bone healing but AM-POC allowed a complete closure of palate cleft and a significantly better palate growth than AM alone after 2 months. AM induced significantly more blood vessels than AM-POC.
Fenelon et al. (2020)	Mice (No. = 6)	Femoral defectDiameter: 1.3 mm	1) Empty defect 2) Fresh AM 3) Cryo AM 4)Lyophilized AM 5) Decellularized + Lyophilized AM	3D X-raysHistology	Covering the defect with lyophilized or decellularized and lyophilized AM significantly enhanced early bone formation. One month after the surgery, the decellularized and lyophilized AM was the only membrane which significantly increased bone formation compared to the defect left empty. Covering the defect with lyophilized or decellularized and lyophilized AM resulted in a significant increase in blood vessels density.

*ADDM, autogenous demineralized dentin matrix; AM, amniotic membrane; Cryo, cryopreserved; DBM, demineralized bone matrix; EPI, epithelial side; HA, Hydro Hydroxyapatite; MBCP, synthetic biphasic calcium phosphate bone substitute; MES, Mesenchymal side; POC, poly(1,8-octamethylene-citrate)*.

**Table 3:** Pre-clinical studies using AM seeded with stromal cells to promote bone regeneration.

**References**	**Animal (No. per condition and per time)**	**Model: Defect localization and size**	**Cells characteristics (*n* = number of cells seeded on AM)**	**Treatments**	**Culture duration before implantation**	**Bone regeneration assessment**	**Results**
Tsugawa et al. (2011)	Mice (No. = 3, 4, 5, and 4)	Calvarial defect4.6 mm diameter	KUSA-A1 cell line (*n* = 7.8 × 10^4^ cells)	1) Empty defect 2) AM 3) AM + injected KUSA-A1 4) AM-KUSA-A1	Up to 20 h	3D X-RayHistology	AM seeded with KUSA-A1 significantly increased bone formation compared to other conditions after 5 weeks.
Semyari et al. (2016)	Rabbit (No. = 1)	Calvarial defect8 mm diameter	Rabbit ADMSC (*n* = 1 × 10^5^ cells per cm^2^)	1) AM 2) PLGA 3) Polyamide 4) AM-ADMSC 5) PLGA-ADMSC 6) Polyamide-ADMSC	6 h	Histology	All seeded scaffolds boosted significantly bone regeneration compared to scaffolds alone after 4 weeks whereas no significant difference was observed after 8 weeks.
Amer et al. (2015)	Dog (No. = 3)	Segmental femoral defectLength: 2 cm	Dog BMSC (*n* = NS)	1) Empty defect 2) AM 3) AM-BMSC	1 week	2D X-rayHistology	AM and AM-BMSCs increased bone healing compared to the empty defect after 6, 12, and 24 weeks.
Wu et al. (2016)	Rat (No. = 5)	Alveolar defectSize: 2.6 × 2.0 × 2.0 mm	Human ADSC (*n* = 3 × 10^5^ cells)	1) Matrigel®- PBS 2) Matrigel®- ADSC 3) AM 4) AM-ADSC	5 days	3D X-Ray	AM and seeded AM significantly induced more bone formation than the two other groups after 29 days without significant difference between AM and seeded AM.
Akazawa et al. (2016)	Mice (No. = 10)	Calvarial defect3.75 mm diameter	Human PDLSC and OB(*n* = 5 × 10^5^ cells)	1) AM-PDLSC 2) AM-OB 3) AM-PDLSC-OB	5–18 h	Histology3D X-Ray	AM-PDLSC-OB significantly enhanced bone regeneration compared to AM seeded with one cell type after 2, 4, and 8 weeks.
Takizawa et al. (2019)	Mice (No.: NS)	Subcutaneous implantation	Human DPSCs (*n* = 1 × 10^5^ cells/mL)	1) AM-DPSCs in osteogenic medium 2) AM-DPSCs in control medium	4 weeks	Histology2D X-ray	AM-hDPSCs in osteogenic medium expressed more bone feature than hAM-hDPSCs in control medium on ectopic site after 4 weeks.
Takizawa et al. (2019)	Rat (No. = 3, 5, and 11)	Alveolar defectSize: NS	Human DPSCs (*n* = 1 × 10^5^ cells/mL)	1) Empty defect 2) AM-DPSCs in control medium 3) AM-DPSCs in osteogenic medium	4 weeks	3D X-Ray	Osteodifferentiated hDPSCs seeded on AM increased significantly alveolar bone formation after 4 weeks.
Sabouri et al. (2020)	Rat (No. = 4)	Calvarial defect6 mm diameter	Human ADSC (*n* = 1 × 10^6^ cells/mL)	1) Empty defect 2) DAM 3) DAM-ADSC 4) MAM 5) MAM-ADSC	NS	Histology3D X-Ray	Both seeded scaffolds significantly enhanced bone regeneration compared to scaffolds alone after 4 and 8 weeks. The best results were achieved by the MAM-seeded scaffold.
Dziedzic et al. (2021)	Rat (No. = 5)	Calvarial defect8 mm diameter	Rat ADSC (*n* = 5 × 10^4^ cells per cm^2^)	1) Empty defect 2) AM 3) AM-ADSC	7 days	Histology3D X-Ray	AM-ADSC significantly enhanced bone regeneration compared to the empty defect after 12 weeks. No significant difference between AM and AM-ADSC.

*ADMSC, adipose-derived mesenchymal stromal cells; ADSC, adipose-derived stromal cells; AM, amniotic membrane; BMSC, bone marrow mesenchymal stromal cells; DAM, decellularized amniotic membrane; DPSCs, dental pulp-derived cells; KUSA-A1, mouse bone marrow derived stromal cells; MAM, mineralized amniotic membrane; NS, not specified; OB, calvarial osteoblasts; PBS, phosphate-buffered saline; PDLSC, periodontal ligament stem cells; PLGA, poly lactic-co-glycolic acid*.

#### Ectopic Sites

Two studies showed the absence of osteoinductive potential of AM for bone regeneration in a subcutaneous ectopic model in mice. Subcutaneous implantation is the simplest experimental model to assess ectopic bone formation, reducing the number of variables involved in bone formation (i.e., stimulating cytokines, bone forming cells, endogenous stem cells, and potentially bone-stimulating mechano-transduction) (Scott et al., 2012). In the first study, AM was assessed either fresh or after being osteodifferentiated *in vitro*, combined or not with a bone substitute (Table 2). Eight weeks after surgery, neither fresh nor osteodifferentiated AM induced ectopic bone formation, whether or not it was associated with the bone substitute (Laurent et al., 2017). The second study investigated the efficacy of dental pulp-derived cells (DPSCs) seeded on AM to induce bone formation (Takizawa et al., 2019) (Table 3). The study compared DPSCs sheets cultured in an osteoinductive or control culture medium. Both sheets were grafted subcutaneously. Four weeks after implantation 2D-Radiography, immunological and histological analysis were performed. Qualitative staining suggested better results with osteodifferentiated DPSCs sheets that showed higher osteocalcin expression, and had higher alkaline phosphatase, von Kossa, and alizarin red staining compared to the sheets cultured in the control medium This study did not show quantitative analysis and did not perform an osteoinductive positive control.

#### Orthotopic Sites

Eight studies assessed the efficacy of AM used alone to regenerate surgical bone loss (Tables 1, 2). Samandari et al. studied the bone remodeling of a jaw mucoperiosteal defect in dogs (Samandari et al., 2011). Covering the defect with AM significantly enhanced bone formation. Tang et al. were the first to assess the use of AM as a barrier membrane for guided bone regeneration (Tang et al., 2018). They showed that the implantation of AM over the defect increased significantly bone formation compared to the femoral defect left empty in rats. This result was corroborated by the enhancement of gene expression connected with cell recruitment and bone remodeling expression. Recently, Sari et al. investigated the efficacy of wrapped AM to promote tibial bone shaft fracture healing in rats (Sar et al., 2019). They observed that wrapping AM circumferentially around the fracture line significantly promoted fracture healing compared to the bone reduction without membrane. The ability of AM to act successfully as a barrier membrane for guided bone regeneration was also supported by another study, which compared AM to a currently used collagen membrane. In their study, Koushaei et al. also observed that bone formation was significantly enhanced when the tibial defect was covered by AM compared to the empty defect in dogs (Koushaei et al., 2018). Additionally, no significant difference in the amount of regenerated bone was evidenced between the commercial collagen membrane and the empty defect, thereby suggesting that AM is more effective than this commonly used collagen membrane to guide bone regeneration. The bone healing potential of AM used as a membrane to cover bone defect was also reported with larger bone defect models. Indeed, two studies showed the efficacy of covering a rabbit segmental bone defect with AM to promote bone regeneration (Ghanmi et al., 2018; Moosavi et al., 2018). Finally, only two studies investigated the influence of AM preservation methods on its bone regeneration potential. One study investigated the impact of cryopreservation on bone regeneration potential of AM compared to fresh AM. The AM cell layer oriented toward the bone with the best regeneration efficiency was also investigated. Results suggested that the use of cryopreserved AM with the mesenchymal side in contact with the calvarial defect was the best condition to favor bone regeneration in mice (Fénelon et al., 2018b). The other study compared four commonly used preservation methods of AM (i.e., fresh, cryopreserved, lyophilized or decellularized, and lyophilized) for GBR procedures in mice tibial defect (Fenelon et al., 2020). Covering the defect with lyophilized or decellularized and lyophilized AM significantly enhanced early bone formation. One month after the surgery, only the decellularized and lyophilized AM significantly promoted higher bone regeneration.

Conversely, three studies suggested that using AM as a filling material, instead of using it as a covering membrane, did not promote bone regeneration but rather delayed it (Ghanmi et al., 2018; Khalil and Melek, 2018; Moosavi et al., 2018). Khalil et al. observed that bone formation was significantly lower when AM was used to fill the femoral defect compared to the empty defect in rabbits (Khalil and Melek, 2018). The two other studies performed a rabbit segmental bone defect and compared, among other things, the efficacy of AM as a membrane covering the defect or filling the bone defect. They both observed a significant higher bone formation when AM was used to cover the defect, whereas no difference was evidenced between the empty defect and the defect filled with AM (Ghanmi et al., 2018; Moosavi et al., 2018).

Only one study used the AM hybridized with a synthetic polymer (poly 1,8-octamethylene-citrate) to create a cell-free, resorbable, tissue engineered graft for complete cleft palate. Li et al. studied palate cleft healing using AM or AM combined with the polymer in rats (Li W. et al., 2019). A complete hard palate reconstruction with a complete bone union was achieved by AM combined with the polymer compared to AM used alone. Unfortunately, this study did not assess healing using the polymer without AM, making thus difficult to draw any conclusion.

Few studies investigated the additional osteoconductive properties of AM associated with a bone substitute. Gomes et al. performed a qualitative histological evaluation that showed a greater amount of bone tissue and a faster bone healing process when AM covered the bone substitute compared to AM alone in a rabbit calvarial defect model (Gomes et al., 2001). Two studies also investigated the potential of AM as a membrane covering a bone substitute and compared it to a currently used collagen membrane (Li et al., 2015; Fénelon et al., 2018b). Li et al. assessed the potential of AM associated with a bone substitute in a rat tibial defect surrounding a cylindrical titanium screw mimicking guiding bone regeneration around a dental implant (Li et al., 2015). The AM seemed to better avoid invasion of fibrous tissue, thus promoting more bone healing than the collagen group. In a previous study, we also compared AM and a commercially available collagen membrane to cover a bone substitute associated or not with the BMP-2 growth factor in a mouse calvarial bone defect model (Fénelon et al., 2018b). However, this study failed to evidence any significant difference between groups without membrane and groups covered by a collagen membrane or AM.

Finally, eight studies proposed the use of AM as a scaffold, which could be seeded by stromal cells before its implantation (Tables 1, 3). We identified seven types of stromal cells, which have been seeded on AM to promote bone regeneration. Among them six were primary cells whereas one study used cell line (Tsugawa et al., 2011). They were cultured on AM for 6 h and up to 4 weeks before its implantation *in vivo*. Tsugawa et al. investigated the potential of using AM as a scaffold seeded with a bone marrow derived stromal cell line prior its implantation in a mouse calvarial bone defect model (Tsugawa et al., 2011). They observed earlier and greater bone regeneration of calvarial defect with the seeded AM. Amer et al. performed qualitative analysis of bone regeneration in a dog femoral segmental bone defect and stated that AM and dog bone marrow mesenchymal stromal cells (BMSCs)-seeded AM enhanced bone healing compared to the empty condition (Amer et al., 2015). They also reported that bone regeneration occurred sooner when AM was seeded with BMSCs before its implantation. However, no quantitative analysis was present in this study to support the results. Three studies investigated the ability of AM to act as a scaffold seeded with adipose derived stromal cells (ADSCs) for bone regeneration. Wu et al. reported that bone regeneration was significantly enhanced when the rat alveolar defect was filled with AM or human ADSCs-seeded AM compared to the empty defect or defect filled with cells (Wu et al., 2016). No statistical difference between AM and seeded AM was evidenced in this study. Dziedzic et al. showed a significant higher bone formation using seeded AM compared the defect left empty (Dziedzic et al., 2021). However, they also failed to evidence significant difference between AM and seeded AM in contrast with the study of Semyari et al. which observed an earlier bone regeneration when rabbit ADSCs-seeded AM was implanted in a rabbit calvarial bone defect (Semyari et al., 2016). However, almost complete defect closure was observed in all experimental groups after 8 weeks. Akazawa et al. investigated the bone regeneration potential of AM seeded with two different stromal cells types (human periodontal ligament stem cells and calvarial osteoblasts) either together or separately (Akazawa et al., 2016). Significant better results were achieved when the two cell types were combined and seeded on AM using a calvarial bone defect in mice. Takizawa et al. compared the suitability of AM seeded with human DPSCs after being cultured *in vitro* in two different conditions (osteogenic or control medium) (Takizawa et al., 2019). X-ray showed a significant bone-like tissue outgrowth when the cell-seeded AM had been cultured in osteogenic medium prior to its implantation in a rat alveolar defect. However, this study lacks histological data to support this result. Finally, Sabouri et al. proposed a novel approach by mineralizing AM before cell-seeding (Sabouri et al., 2020). They stated that the mineralized and seeded AM promoted higher bone regeneration than conventional seeded AM.

### Use of AM to Promote Bone Regeneration in Clinical Studies

The seven included clinical studies on the osteogenic potential of the membrane were all performed in the field of oral and maxillo-facial surgery (Table 4). They used AM as an allograft membrane and AM was mostly processed as a lyophilized membrane (*n* = 6), otherwise the membrane was decellularized then lyophilized (*n* = 1) (Table 1) (Akhlaghi et al., 2019). AM was used to cover a bone substitute in six studies.

**Table 4:** Clinical studies using amniotic membrane to guide bone regeneration.

**References**	**No. = (Patients per group)**	**Indication**	**Treatments**	**Evaluation methodology**	**Results**
Kothiwale et al. (2009)	No. = 10	Periodontal furcation defect (Grade II)	1) DFDBA + AM 2) Bio-oss®+ AM	Measurement of CAL and PPD 2D Radiography	Significant improvement of parameters from baseline to 9 months in both groups without significant differences between groups.
Kiany and Moloudi (2015)	No. = 10	Periodontal pockets	1) Bio-oss®+ AM 2) Bio-oss®+ Collagen membrane (Bio-gide®)	Measurement of CAL, PPD, GR, and probing bone	Significant improvement of parameters from baseline to 6 months in both groups. No significant differences between the two groups after 6 months.
Kumar et al. (2015)	No. = 27	Periodontal pockets	1) G-graft® 2) G-graft® + AM	Measurement of CAL, PPD, and inflammatory 2D Radiography	AM significantly increased bone fill and CAL after 6 months.
Sali and Pauline George (2016)	No. = 10	Periodontal pockets	1) DFDBA 2) DFDBA + AM	Measurement of CAL, PPD, and GR 3D Radiography	Significant improvement of parameters from baseline to 12 months in both groups. No significant differences between the two groups at 12 months.
Pajnigara et al. (2017)	No. = 20	Periodontal furcation defect (Grade II)	1) DFDBA 2) DFDBA + AM	Measurement of CAL, PPD, GR, and horizontal probing depth 3D Radiography	AM increased significantly bone fill and CAL at 6 months. AM reduced significantly PPD and GR at 6 months.
Kaur and Bathla (2018)	No. = 15	Periodontal furcation defect (Grade II)	1) PRF 2) PRF + AM	Measurement of CAL and PPD 3D Radiography	AM significantly increased bone fill and CAL at 6 months. AM significantly reduced PPD at 6 months.
Akhlaghi et al. (2019)	No. = 9	Jaw-bone defect	1) NBBM+ bone autograft + AM 2) NBBM + bone autograft + AM loaded with BFSCs	3D RadiographyHistology	The mean increase in bone width was significantly greater in the AM + BFSCs group at 5 months.

*AM, amniotic membrane; BFSCs, buccal fat pad-derived stromal cells; CAL, clinical attachment level; DFDBA, demineralized freeze dried bone allograft; GR, gingival recession; NBBM, natural bovine bone mineral; PPD, probing pocket depth; PRF, platelet-rich fibrin*.

Kaur et al. showed that bone formation was enhanced when periodontal furcation defects were filled with a platelet-rich fibrin (PRF) membrane then covered by AM (Kaur and Bathla, 2018). However, it is more widely recognized to use a bone substitute rather than a PRF membrane as a filling material to treat periodontal defects. The first randomized clinical trial investigating the potential of AM covering a bone substitute to guide bone regeneration was performed by Kothiwale et al. (2009). This study compared two groups, which both used AM and assessed its efficacy to cover either xenogeneic or allogenic bone grafts for periodontal furcation defect treatment. They stated that this association significantly improved bone formation. Unfortunately, there was no control group without the membrane to specifically highlight its effect. Three studies were then performed to compare bone formation with or without AM in the field of periodontal surgery. Kumar et al. filled periodontal pockets with hydroxyapatite bone graft, which was covered or not by AM (Kumar et al., 2015). They showed a significant improvement of clinical and radiological parameters using AM to cover the bone substitute. This was corroborated by Pajnigara et al. who displayed similar results for the treatment of periodontal furcation defect (Pajnigara et al., 2017). The association of a bone allograft with AM resulted in a more significant quantity of regenerated tissue than the bone substitute alone. However, contradictory results were obtained by Sali et al., which observed similar improvement of periodontal pockets regeneration using or not AM to cover the bone substitute (Sali and Pauline George, 2016). This study was conducted on a smaller sample size than the two above-mentioned studies. Interestingly, one study compared AM to a commercial resorbable collagen membrane commonly used in oral surgery (Kiany and Moloudi, 2015). Both membranes were applied over a bone xenograft to treat periodontal pockets. No significant difference was observed between this resorbable collagen membrane, gold standard for this application, and AM after 6 months, thereby demonstrating that AM could be a promising alternative.

Finally, one clinical study investigated the potential of AM to favor large bone defect healing in jaws. Akhlaghi et al. used a decellularized AM in combination with autologous buccal fat pad-derived stem cells to cover large bone grafts prior to implant placement (Akhlaghi et al., 2019). They reported a better healing of jaw-bone defect when the bone graft was covered with AM loaded with stem cells compared to AM alone. However, there was no membrane-free group from which to draw conclusions about the role of AM in the healing process.

### Use of Amniotic Cells to Promote Bone Healing

Ten studies assessed *in vivo* the potential of AECs and AMSCs as a source of cells for bone regenerative therapies (Table 5). A bone substitute was used as a scaffold in five studies. The cell culture duration after seeding the scaffold and before its implantation was 3–42 days.

**Table 5:** Pre-clinical studies using amniotic membrane derivated stromal cells for bone regeneration.

**References**	**Animal (No. per condition and per time)**	**Model: Defect localization and size**	**Amniotic cells characteristics (*n* = number of cells seeded on scaffold)**	**Scaffold (*t* = culture duration before implantation)**	**Treatments**	**Evaluation methodology**	**Results**
Mattioli et al. (2012)	Sheep (No. = 2)	Tibial defectDiameter: 3 mm	oAECs (2 × 10^6^ cells)	Fibrin glue (Tissuecol) (no culture)	1) Tissuecol 2) Tissuecol + oAECs	Histology	Bone deposition was only observed in oAECs-transplanted defects after 45 days.
Tsuno et al. (2012)	Rat (No. = 3)	Calvarial defectDiameter: 5 mm	hAMSCs (1 × 10^7^ cells/mL)	β-TCP (*t* = NS)	1) β-TCP 2) β-TCP + hAMSCs	Histology	hAMSCs seeded scaffold showed immature bone deposition at 6 weeks and mature bone areas at 12 weeks.
Barboni et al. (2013)	Sheep (No. = 3)	Sinus augmentation	oAECs (1 × 10^6^ cells)	HA/β-TCP (*t* = 3 days)	1) HA/β-TCP 2) HA/β-TCP + oAECs	Micro-CTHistology	oAECs seeded scaffold displayed significant earlier bone formation and maturation at 45 days and induced significantly more bone deposition at 90 days.
Chen et al. (2014)	Mice (No. = 6)	Subcutaneous	hAMSCs (5 × 10^4^ cells/mL)	CultiSpher S: Porcine gelatin microcarriers (*t* = 28–42 days)	1) CultiSpher S+ hAMSCs (no perfusion) 2) CultiSpher S+ hAMSCs (1 week perfusion) 3) CultiSpher + hAMSCs (2 weeks perfusion)	Micro-CTHistology	Perfusion culture system increased mineralized matrix after 6 and 12 weeks Perfusion significantly enhanced vessel density.
Jiawen et al. (2014)	Rat (No. = 4)	Alveolar defectSize: NS	hAECs (1.5 × 10^6^)	β-TCP (*t* = 3 days)	1) β-TCP 2) β-TCP + hAECs	Micro-CTHistology	hAECs seeded scaffold significantly increased bone formation at 4 and 8 weeks postoperatively. hAECs seeded scaffold showed a significantly delayed macrophage response.
Si et al. (2015)	Mice (No. = 3)	Subcutaneous	hAECs (1.5 × 10^6^)	β-TCP (*t* = 3 days)	1) β-TCP 2) β-TCP + hBMSCs 3) β-TCP + hAFMSCs 4) β-TCP + hAECs	HistologyImmunohisto-chemistry	No sign of mineralization in all groups 1 month after implantation. OPN and OCN were expressed at a higher level with the seeded scaffolds.
Rameshbabu et al. (2016)	Rabbit (No. = 5)	Osteochondral defectDiameter: 4 mmDeep: 5 mm	hAMSCs (NS)	PEMS (7 days)	1) Empty defect 2) PEMS 3) PEMS + hAMSCs	Histology	hAMSCs seeded scaffold seemed to induce higher bone formation and osteochondral regeneration 60 days post-implantation.
Jiang et al. (2020)	Rabbit (No. = 3)	Calvarial defectDiameter: 10 mm	hAMSCs (5 × 10^6^ cells/mL)	Fibrinogen solution (NS)	1) Fibrin gel 2) Bio-oss 3) hAMSCs 4) Bio-oss + hAMSCs	Micro-CTHistologySequential fluorescent labeling	Bone regeneration was significantly higher in groups 3 and 4 after 4 and 12weeks. Fluorescent labeling was significantly higher in group 4 after 3, 6, and 9 weeks. Vessels-like structure are significantly higher in presence of hAMSCs.
Li et al. (2020)	Rat (No. = 3)	Calvarial defectDiameter: 3 mm	hAMSCs (1 × 10^6^ cells/mL)	Fibrin (NS)	1) Fibrin 2) Fibrin + hAMSCs	Micro-CTHistology	hAMSCs seeded scaffold induced significantly higher bone formation after 8 weeks.
Datta et al. (2021)	Rabbit (No. = NS)	Tibial defectDiameter: 2.5 mmDeep: 2 mm	hAMSCs (1 × 10^6^ cells/mL)	Hydrogel hybride (DBM + chitosan)	1) Empty defect 2) Hydrogel 3) Hydrogel + hAMSCs	Micro-CTHistology	Bone regeneration was significantly higher using hydrogel + hAMSCs after 4 and 8 weeks. hAMSCs seeded scaffold showed more vascular structure after 4 weeks compared to the two other groups.

*β-TCP, β-tricalcium phosphate; DBM, demineralized bone matrix; HA, hydroxyapatite; hAECs, human amniotic epithelial cells; hAMSCs, human amniotic mesenchymal stromal cells; NS, Not specified; oAECs, ovine amniotic epithelial cells; OCN, osteocalcin; OPN, osteopontin; PEMS, Placenta-derived Extracellular Matrix Sponge*.

#### Ectopic Sites

A mouse ectopic model was used in two studies to assess the osteoinductive potential of amniotic membrane cells seeded on a biomaterial. These are the only studies that clearly specified that AECs and AMSCs were first cultivated in an osteoinductive medium before being seeded on the scaffold and then implanted. Chen et al. (2014) created an *in vitro* perfusion culture system to irrigate the AMSCs-seeded scaffold before its implantation *in vivo* and compared it to the same AMSCs-seeded scaffold, which was only cultured in osteogenic medium without being followed by perfusion. Better results were achieved using the perfusion culture system. Si et al. compared the osteogenic potential of AECs seeded on a bone substitute to two other types of stromal cells including gold standard human BMSCs (Si et al., 2015). No subcutaneous bone formation was observed whatever the condition tested. However, bone associated extracellular matrix proteins seemed to show higher staining with the seeded scaffolds.

#### Orthotopic Sites

Eight studies investigated the potential of AECs or AMSCs seeded on a scaffold to regenerate bone defects (Table 5). Three studies assessed *in vivo* the use of AECs seeded on a scaffold to the scaffold implanted alone for bone regeneration. They all reported that AECs display osteogenic potential. Mattioli et al. performed a qualitative histological analysis in a sheep tibial defect model and observed that ovine AECs-transplanted defects were filled with newly deposited bone whereas only fibrous tissue was observed in the fibrin glue control defects (Mattioli et al., 2012). In the two other studies, AECs were seeded on an osteoconductive scaffold (i.e., bone substitute). Barboni et al. reported the ability of ovine AECs seeded scaffold to significantly promote bone regeneration and maturation in sheep sinus augmentation surgery compared to the scaffold without cells (Barboni et al., 2013). This was corroborated by Jiawen et al. which showed a significant increase in bone formation and a decrease in inflammatory reaction using human AECs seeded scaffold compared to the non-seeded scaffold after 1 and 2 months in rat alveolar defects (Jiawen et al., 2014).

Five studies assessed *in vivo* the potential of AMSCs seeded scaffold to promote bone regeneration. Tsuno et al. stated that more bone deposition was observed with human AMSCs seeded on an osteoconductive scaffold compared to the non-seeded scaffold in a rat calvarial defect model (Tsuno et al., 2012). Rameshbadu et al. investigated the osteogenic potential of human AMSCs in an osteochondral bone defect in rabbits (Rameshbabu et al., 2016). They observed higher osteochondral bone formation with the AMSC seeded scaffold. Two other studies also reported significantly higher bone regeneration using hAMSCs seeded scaffold compared to the non-seeded scaffold (Li et al., 2020; Datta et al., 2021). Only one study compared a “hAMSCs seeded scaffold” to the same cells implanted alone without the scaffold. Both groups containing hAMSCs (with or without the scaffold) showed significantly higher bone formation in a rabbit calvarial defect (Jiang et al., 2020).

### Commercialized AM-Derived Products for Bone Regeneration

Four studies used commercialized AM-derived products to promote bone healing in pre-clinical and clinical settings (Table 6). Two studies reported the use of the commercial preparation NuCel® associated with a bone autograft. NuCel® is an amniotic suspension allograft, derived from human AM and amniotic fluid cells that is cryopreserved to maintain bioactivity. Starecki et al. assessed the bone regeneration potential of NuCel® mixed with a bone graft in a rat segmental bone defect (Starecki et al., 2014). Bone graft preparation mixed with NuCel® did not improve bone formation compared to the bone graft group after 6 weeks. One clinical study proposed to perform lumbar interbody fusion in patients using bone allograft mixed with NuCel® (Nunley et al., 2015). This retrospective analysis showed that 97.4% of one-level patients and 100% of two-level patients were clinically fused. These results were similar to those found in the literature for conventional lumbar interbody fusion procedure. Konofaos et al. investigated the potential of another commercialized preparation, which is the AmnioMTM®, to promote bone healing of rat calvarial defects (Konofaos et al., 2015). This product is an injectable gel obtained after grinding cryopreserved amnion. In this study, the AmnioMTM® was mixed with a bone substitute before grafting. Contradictory findings were obtained after 1 month of implantation. Micro-CT data showed that the addition of AmnioMTM® to the bone substitute did not significantly increase new bone formation compared to the grafted bone substitute alone, though statistical difference was observed with histomorphometric analysis. Finally, Burdette et al. reported the use of a secretome (ST266, Noveome Biotherapeutics) derived from cultured human amniotic cells for bone formation in a rat calvarial defect (Burdette et al., 2017). The secretome is a liquid suspension containing biomolecules released by amniotic cells. Micro-CT analysis showed no significant difference in terms of bone volume regenerated, whereas the secretome significantly enhanced bone density.

**Table 6:** Use of commercialized AM-derived products for bone regeneration.

**References**	**Subject (No. per condition)**	**Model**	**Consistency**	**Treatments**	**Evaluation methodology**	**Results**
Starecki et al. (2014)	Rat (No. = 14)	Segmental femoral defect8 mm	Liquid suspension	1) Empty defect 2) Bone substitute 3) Bone substitute mixed with NuCel®	2D X-Ray Histology	No significant differences in bone formation between bone graft alone and bone graft mixed with NuCel®after 6 weeks.
Konofaos et al. (2015)	Rat (No. = 5)	Calvarial defect10-mm diameter	Injectable gel	1) DBM 2) DBM + amnioMTM®	Micro-CT Histology	Micro-CT: No significant differences in bone formation between both groups after 4 weeks. Histology: A significantly higher mean percent of new bone in the defect for the DBM group as compared with the DBM + AmnioMTM group after 4 weeks.
Nunley et al. (2015)	Human (No. = 72)	Lumbar interbody fusions	Liquid suspension	1) Bone allograft mixed with NuCel®	2D X-ray Micro-CT	Allograft + NuCel® demonstrated high fusion rates after a minimum of 12 months post-operation.
Burdette et al. (2017)	Rat (No. = 5)	Calvarial defect8-mm diameter	Liquid suspension	1) Collagen sponge 2) Collagen sponge + Secretome biotherapeutic (ST266) (Noveome Biotherapeutics)	Micro-CT Histology	No significant differences in bone volume formation between both groups. Secretome significantly enhanced angiogenesis after 4 weeks and bone density after 4 and 12 weeks.

*DBM, demineralized bone matrix*.

## Discussion

This article aimed at reviewing the bone regenerative medicine approaches of AM and its derivatives as well as their limitations.

We outlined that three categories of AM-derived products were mainly used for bone regeneration: (i) AM (ii) AM cells, and (iii) commercialized AM-derived products. Most of the included studies investigated the use of AM as a membrane for bone regeneration. AM is a promising natural allogenic biomaterial which is widely available without ethical concerns. Moreover, compared to collagen or synthetic membranes commonly used for guided bone regeneration procedures, AM displays several biological properties making it very attractive for this field (Gindraux et al., 2017; Fénelon et al., 2018a; Aprile et al., 2020; Gulameabasse et al., 2020). AM promotes epithelialization, namely by excreting epithelial growth factor (EGF) (Jin et al., 2016) and has also the ability to modulate angiogenesis (Niknejad et al., 2013). In addition, AM has antimicrobial activity by expressing natural antimicrobial molecules such as β-defensins and elafin (King et al., 2007) and shows anti-fibrotic properties, due to secretion of tissue inhibitors of metalloproteinase (TIMPs) and the down-regulation of transforming growth factor beta (TGF-β) in fibroblasts, which is responsible for their activation, thereby reducing the risk of adhesion and scarring (Lee et al., 2000; Ricci et al., 2013). These biological properties, as well as mechanical properties of AM, might be modulated or affected by the preservation process (Yazdanpanah et al., 2015; Fenelon et al., 2019).

Thanks to its low immunogenicity, all but two studies (Amer et al., 2015; Semyari et al., 2016) used human AM either as a xenograft or as an allograft. AM is known to be an immune-privileged tissue and to contain some immunoregulatory factors, including HLA-G (an immunosuppressive factor) and Fas ligand (Kubo et al., 2001). This effect is also supported by the low/absent level of expression of HLA class I molecules and the absence of HLA class II molecules (Ilancheran et al., 2009), avoiding allograft or xenograft rejection of human AM. We identified several methods of preparation of AM in pre-clinical studies. AM was used either fresh, cryopreserved, or lyophilized (i.e., freeze-dried). Moreover, preserved AM has been applied intact, de-epithelialized or decellularized (without epithelial and mesenchymal stromal cells). Unfortunately, none of the included studies has simultaneously compared these preservation methods, making it difficult to conclude if there is an optimal preservation method of AM for bone regeneration. The wide heterogeneity of animal models (species, localization and size of the defect) increases the difficulty of comparing these conditioning methods. One study compared four commonly used preservation methods of AM (i.e., fresh, cryopreserved, lyophilized, and decellularized-lyophilized AM) using the same animal diaphyseal bone defect model in mice (Fenelon et al., 2020). No significant difference was observed between the empty defect and the defect covered either by fresh or cryopreserved AM, whereas lyophilized and decellularized-lyophilized AM significantly enhanced early bone regeneration. Interestingly, both methods are used when AM is applied to guide bone regeneration in humans (Kothiwale et al., 2009; Kiany and Moloudi, 2015; Kumar et al., 2015; Sali and Pauline George, 2016; Pajnigara et al., 2017; Kaur and Bathla, 2018; Akhlaghi et al., 2019). This result can be explained by the safe and long term storage of samples at room temperature allowed by lyophilization (Rodríguez-Ares et al., 2009), which could also be easily followed by gamma irradiation to sterilize the membrane (Gindraux et al., 2013). Dehydration is another reported preservation procedure of AM (Dadkhah Tehrani et al., 2020). Although this method is used for gingival recession or mucosal defect treatments in oral surgery (Fénelon et al., 2018a), none of the studies included in the present review used dehydrated AM for bone regeneration. Most of the studies which investigated the ability of AM to act as a scaffold seeded with stem cells before its implantation in bone defects used de-epithelialized or decellularized AM (Tsugawa et al., 2011; Akazawa et al., 2016; Semyari et al., 2016; Wu et al., 2016; Takizawa et al., 2019). This could be related to the exposure of the basement membrane caused by the de-epithelialization or decellularization process, thereby promoting AM ability to favor cell adhesion and proliferation (Koizumi et al., 2007; Riau et al., 2010; Salah et al., 2018; Fenelon et al., 2019). We believe that the decellularization process induce less variability and is more reproductible than a de-epithelialization which is often required a scraping step that is operator-dependent (Fenelon et al., 2019). Most of the included studies pointed that AM is mainly applied as a single layer. Few studies suggested to create a multilayered AM (Li et al., 2015; Dziedzic et al., 2021). This might enhance its thickness that initially ranges from 0.02 to 0.5 mm (Bourne, 1962). In this respect, a recent study demonstrates that AM epithelium is not single-layered in the different regions (Centurione et al., 2018), thus AM from the central region could be more appropriate to transplant when a greater thickness is requested. However, while AM is a thin membrane, several authors reported that AM is easy to handle and to adapt to the surgical site (Tsuno et al., 2014; Kiany and Moloudi, 2015; Fénelon et al., 2018a). Our results also showed that AM was mainly used as a barrier membrane covering bone defects. Otherwise, AM was applied as a filling material inside the bone defect area. We observed that better results were achieved when AM was used to cover the defect rather than as a filler since grafting the AM into the bone defect seems to hinder the bone regeneration process (Ghanmi et al., 2018; Khalil and Melek, 2018; Moosavi et al., 2018). This allows us to conclude that it is better to apply AM over the defect acting as a barrier membrane, thereby preventing the bone defect area from fibrous tissue invasion. This occlusive function is one of the main characteristics required for membranes used in guided bone regeneration procedure (Aprile et al., 2020). We did not find information concerning the space maintenance ability of AM which is another needed criteria for membranes in guided bone regeneration procedures (Naung et al., 2019). However, unlike some animal studies, almost all clinical studies used a bone substitute that was then covered by AM (Kothiwale et al., 2009; Kiany and Moloudi, 2015; Kumar et al., 2015; Sali and Pauline George, 2016; Pajnigara et al., 2017; Akhlaghi et al., 2019). Maintaining the space with a bone substitute to support the membrane is often mandatory with biological membrane which lacks mechanical properties to avoid membrane collapse within the defect during the healing (Bunyaratavej and Wang, 2001; Soldatos et al., 2017). Only few studies compared AM with conventional membranes used for guided bone regeneration procedures (Kiany and Moloudi, 2015; Li et al., 2015; Fénelon et al., 2018b; Koushaei et al., 2018). It was then compared to collagen membranes which are the resorbable natural membranes most used for guided bone regeneration (Bunyaratavej and Wang, 2001; Aprile et al., 2020). Preserved AM (cryopreserved, decellularized or lyophilized) seemed to be at least as efficient as these conventional and commercially available membranes. AM seemed superior in avoiding fibrous tissue invasion that could be linked to its anti-fibrotic properties. However, it is noteworthy that very few studies have focused on the resorption rate of AM even though it is a parameter often criticized in conventionally used resorbable membranes. When mentioned, the resorption duration of AM ranged from 1 to 2 months (Fénelon et al., 2018b; Tang et al., 2018). Thanks to its biological properties and growth factors content, AM has become a very attractive bioactive membrane for bone regeneration. Similarities between AM and the induced membrane (Masquelet technique) have even been shown (Gindraux et al., 2017). The induce membrane is an autologous biological membrane currently used to treat segmental long bone defects and required a two-step surgical procedure. Both membranes share similar proteins components, have comparable thickness, contain growth factors such as VEGF or TGF-β1 and express anti-inflammatory proteins (Pelissier et al., 2004; Grzywocz et al., 2014; Gindraux et al., 2017; Litwiniuk et al., 2018). The use of AM as an existing biological membrane could simplify this approach into a one-step procedure, which would reduce risk to the patient and surgical costs (Fénelon et al., 2021). Besides, the absence of calcification detected in an ectopic model, namely after subcutaneous implantation of AM (Laurent et al., 2017), suggest the immune tolerance of AM (Wilshaw et al., 2008). It is assumed that tissue rejection involves calcinosis and structural degeneration of the graft that are caused by immune response against donor cells (Muratov et al., 2010). In this systematic review, most of the experiments assessing bone regeneration using AM were performed in an orthotopic site, mimicking the mechanical and chemical influences that bone receives in clinical applications. In animal studies, the orthotopic evaluation of AM was mainly performed using cranial, maxillary or mandibular bone defects, otherwise long bone defects were used. This is consistent with clinical studies which are all dedicated to oral and maxillofacial surgery.

To further enhance the osteogenic potential of AM and develop a qualifiable functional engineered product, some authors suggested to seed stem cells on AM before its implantation. These studies showed that AM displays the ability to act as an appropriate natural scaffold, on which stem cells can grow and differentiate toward the osteogenic lineage. However, it is difficult to draw any conclusions from these studies and to discern the effect of AM from the stromal cells used on bone regeneration. First, contradictory results were observed. Indeed, two studies reported an enhancement of bone regeneration using either AM or seeded AM (Amer et al., 2015; Wu et al., 2016), suggesting that using stromal cells seeded on AM did not further enhance bone regeneration, whereas an increase in bone regeneration was reported with seeded AM compared to AM in three other studies (Tsugawa et al., 2011; Semyari et al., 2016). Furthermore, only one study was conducted using a defect filled with “stromal cells only” as a control group (Wu et al., 2016), whereas all other studies did not compare AM and seeded AM with the condition “stromal cells only,” thereby making it impossible to conclude on the action of stromal cells or AM. The last point that prevents a conclusion from being drawn is related to the fact that five different cell types were used with a wide heterogeneity of culture duration on AM before implantation (from 6 h to 4 weeks). Finally, one clinical trial successfully used AM loaded with buccal fat pad-derived stromal cells (BFSCs) to cover jaw-bone grafts. Among the various sources of mesenchymal stromal cells proposed in bone regenerative medicine, BFSCs display many advantages. They are isolated from the intraoral sources of buccal fat pad that ensures low morbidity after retrieval. BFSCs are thus an easily accessible source of autologous stromal cells which can be readily used for regeneration of craniofacial bone defects (Khojasteh et al., 2019; Meshram et al., 2019). Besides, BFSCs display similar expression level of RUNX-2, osteopontin, osteocalcin, and ALP activity as BMSCs (Ardeshirylajimi et al., 2015), which are currently used for bone tissue engineering approaches (Lin et al., 2019; Kangari et al., 2020).

Various cell types have already been investigated for cell-based bone tissue engineering approach, such as mesenchymal stromal cells (especially bone marrow mesenchymal stromal cells and adipose-derived stromal cells), induced pluripotent stem cells or differentiated osteoblasts (Zhang et al., 2012). This systematic review highlighted the potential of AM-derived stromal cells for bone regenerative medicine. AM is an attractive reserve of two pluripotent cell types for tissue engineering: AECs and AMSCs (Parolini et al., 2009). Both of them have demonstrated their ability to differentiate into various cell types *in vitro* including osteogenic cells (Ilancheran et al., 2007; Parolini et al., 2009). Besides, AECs and AMSCs can be used safely. Unlike embryonic stem cells or induced pluripotent stem cells, the *in vivo* teratoma formation and tumorigenicity of AECs and AMSCs have not been reported (Kang et al., 2012b; Rennie et al., 2012). Here, we showed their effectiveness to promote bone healing in a supportive environment. Indeed, the adjunction of AM-derived cells on a scaffold, namely a bone substitute or fibrin-based scaffold, implanted in a bone defect systematically increased bone formation compared to the same scaffold implanted alone. However, most of included studies showed limited size-samples. It is also not possible to draw conclusions regarding the superiority of one of the two cell types (AECs or AMSCs) as they have never been directly compared in the same study. Besides, comparative studies with the most widely studied sources of mesenchymal stromal cells used in bone tissue engineering, such as bone marrow or adipose tissue (Kangari et al., 2020), are necessary to confirm these promising results. Finally, one clinical trial is underway to evaluate the efficacy and safety of human AECs transplant in non-union of limb fracture patients (NCT03031509).

Few studies investigated the use of commercialized AM-derived products for bone repair (Starecki et al., 2014; Konofaos et al., 2015; Nunley et al., 2015). Pre-clinical studies showed inconclusive results and the only clinical trial was conducted without any control group. It is noteworthy that they all are injectable products used as bone filling materials. Regarding their authorization, even NuCel product is described as being defined by US Food and Drug Administration (FDA) 21 CFR Part 1271, these three commercialized products are currently considered “investigational” (SURG.00011, 2020). We did not find clinical studies using commercial patented AM for guided bone regeneration.

Finally, there were few limitations related to the present study that must be mentioned. Firstly, we observed substantial heterogeneity across the methodology of the selected studies, thereby making it difficult to compare studies. Moreover, most of the included articles showed low level of evidence due to the limited number of animals or patients included per condition and the lack of quantitative analysis or statistical significance. Another identified drawback of this systematic review was the higher number of animal studies included compared to clinical studies across the selected studies. Most of the included studies also lack data to support the benefit of AM anti-fibrotic and angiogenic properties in the bone regeneration process. However, this systematic review tried to provide some evidences on the regenerative potential of AM and AM-derived products *in vivo* and we proposed a clear guidance to perform further studies with higher level of impact (Table 7).

**Table 7:** Strategies suggestion to perform a study on AM or AM derived products in the field of bone regeneration.

**AM use strategy**	**Preparation method**	**Disposition**	**Use**	**Study design**	**Expected results**
AM as a membrane for GBR procedure	Lyophilization or Decellularization + Lyophilization of AM	Over the defectOne layer	Alone or associated with a bone substitute	1. Defect 2. Defect + AM 3. Defect + bone substitute 4. Defect + bone substitute covered by AM 5. Defect + bone substitute covered by conventional membrane use for GBR	- Orthotopic bone regeneration supported by quantitative analysis of 3D-Radiography and histological analysis - Angiogenic and anti-fibrotic effect of AM and its derivatives supported by histological and/or immunohistochemical analysis - Statistical significance
AM as a scaffold for bone tissue engineering construct	Decellularization of AM	Inside or over the defect	Seeded with human mesenchymal stromal cells	1- Defect 2- Defect + cells 3- Defect + AM 4- Defect + AM-seeded cells	
AM cells-based strategies (AECs or AMSCs) for bone tissue engineering construct	AM-cells cultured in basal or osteogenic medium	Inside the defect	Seeded on a bone substitute	1- Defect 2- Defect + AM cells 3- Defect + bone substitute 4- Defect + AM cells seeded on the bone substitute 5- Defect + hADSCs or hBMSCs seeded on the bone substitute	

*hADSCs, human adipose stromal cells; hBMSCs, human bone marrow stromal cells; GBR, guided bone regeneration*.

## Conclusion

The AM and its derivatives are an attractive source of biological tissue and stromal cells for bone regeneration. Thanks to its low immunogenicity, AM and its derivatives could be used either as a xenograft or as an allograft. The lyophilized or decellularized-lyophilized AM are a promising alternative to the commercial membranes used for guided bone regeneration procedures and achieved satisfactory outcomes in oral and maxillofacial surgery. AM is mainly applied as a single layer and provide better results when used as a membrane covering the defect rather than as a filling material. It is better to decellularize AM to enhance its potential to act as a natural scaffold seeded with primary cells before its implantation in bone defects. AM-derived stromal cells also showed their potential to be used successfully in the field of bone regenerative medicine. For this purpose, they have to be seeded on a scaffold, namely a bone substitute. Studies investigating the potential of commercialized AM-derived products were too limited and further studies are required to draw some conclusions.

## Data Availability Statement

The original contributions presented in the study are included in the article/supplementary material, further inquiries can be directed to the corresponding author.

## Author Contributions

MF, FG, and J-CF contributed to conception and design of the study. ME wrote the first draft of the manuscript. HK, SW, and RD wrote sections of the manuscript. All authors contributed to manuscript revision, read, and approved the submitted version.

## Conflict of Interest

The authors declare that the research was conducted in the absence of any commercial or financial relationships that could be construed as a potential conflict of interest.
